# Molecular diagnosis of *Fusarium guttiforme *and Pineapple mealybug wilt-associated virus

**DOI:** 10.1186/1753-6561-8-S4-P121

**Published:** 2014-10-01

**Authors:** Lorena Carnielli, Walkíria Andrade Amorim, Aline Vaz, Patricia Machado Bueno Fernandes, José Aires Ventura

**Affiliations:** 1 Laboratório de Biotecnologia Aplicada ao Agronegócio - Universidade Federal do Espírito Santo, Vitória, ES, 29040-090, Brazil; 2 INCAPER - Instituto Capixaba de Pesquisa, Assistência Técnica e Extensão Rural, Vitória, ES, 29050-790, Brazil

## Background

The state of Espirito Santo has in the fruit growing, one of its main economical activities. One of them, the pineapple growth, represents a good economic potential to increase income and job opportunities in the state. However, diseases such as fusariosis and mealybug wilt of pineapple are responsible for the yield losses of up to 80% [1]. The mealybug wilt of pineapple is caused by the complex Pineapple mealybug wilt-associated virus (PMWaV-1, PMWaV-2, PMWaV-3 and PMWaV-5), a single strand RNA [2], while fusariosis on pineapple is caused by the fungus *Fusarium guttiforme *[3]. This study aimed to develop methodologies for detection of these etiological agents in the pineapple plant.

## Methods

For the analyzes, healthy and diseased pineapple plant were collected at the Experimental Farm of Incaper in the city of Sooretama, in the state of Espirito Santo. The samples were submitted to a superficial disinfection process, followed by nucleic acid extraction protocols with Trizol^® ^and SDS buffer [4]. The nucleic acids were treated with DNAse and Reverse Transcriptase enzymes for samples infected with PMWaV. A RNAse enzyme was used for samples infected with *F. guttiforme*. The diagnosis was realized by molecular biology techniques, conventional PCR and real time PCR with specific primers for each strain of PMWaV [5] and H3 primer for *F. guttiforme*. The samples were visualized by electrophoreses on 1% agarose.

## Results and conclusions

Through these techniques were diagnosed the *F. guttiforme *in fruits, and detected the PMWaV in different plant organs. Conventional PCR does not proved to be a good diagnostic tool due to poor sensitivity, the need for visualization on gels and the high risk of contamination. The technique of Real Time PCR can generated diagnosis with greater sensitivity, reproducibility, accuracy and speed and allowed the detection in diseased plants and plants considered healthy. Therefore, this study created molecular tools for a precise and rapid diagnosis of the etiological agents of the fusariosis disease and mealybug wilt of pineapple that allow indexing plants and propagative material the spread of disease to new areas. It is hoped that this study contribute to raising the standards of quality and competitiveness of Brazilian fruit growing, especially pineapple, the level of excellence required by national and international markets, as well as implement actions for incorporation of technological methods, techniques and processes based mainly on concepts of integrated disease management and food security, with a view to the expansion of this crop, increased productivity and income generation for farmers.
